# Papillary Fibroelastoma as a Cause of Cardiogenic Embolic Stroke in a *β*-Thalassemia Patient: Case Report and Literature Review

**DOI:** 10.1155/2017/8185601

**Published:** 2017-03-26

**Authors:** Re-I Chin, John J. Monda, Maulik Sheth, William Ogle, Gloria Merenda, Debapriya De

**Affiliations:** ^1^Saint Louis University School of Medicine, 1402 S Grand Blvd, Saint Louis, MO 63104, USA; ^2^Department of Internal Medicine, SSM St. Mary's Health Center, 6420 Clayton Rd, Richmond Heights, MO 63117, USA; ^3^Department of Cardiothoracic Surgery, SSM St. Mary's Health Center, 6420 Clayton Rd, Richmond Heights, MO 63117, USA; ^4^Department of Pathology, SSM St. Mary's Health Center, 6420 Clayton Rd, Richmond Heights, MO 63117, USA

## Abstract

We describe a case of a young male without stroke risk factors who presented with a sudden onset of left-sided weakness, left hand numbness, and left eye blurriness. CT scan of the head without contrast and diffusion-weighted MRI of the brain with contrast revealed an ischemic stroke in the right middle cerebral artery distribution. Transesophageal echocardiography (TEE) revealed a mobile pedunculated mass on the posterior surface of the mitral valve. This mass was resected and pathology showed a cardiac papillary fibroelastoma (CPFE), which was determined to be the cause of the patient's cardioembolic stroke. Further workup also found that patient had microcytic anemia secondary to *β*-thalassemia intermedia, a rare hematologic disorder due to defective hemoglobin synthesis. Recently, another case report suggested *β*-thalassemia major may underlie the pathogenesis of CPFE. *β*-Thalassemia major causes a state of chronic inflammation and endothelial damage, which can mediate CPFE formation. Based on literature review, this is the first case report of a CPFE in a patient with *β*-thalassemia intermedia. This hypothesis-generating case report calls attention to the need for elucidating the relationship between CPFE and *β*-thalassemia in future studies to better understand the diagnosis and management of a rare cardiac tumor.

## 1. Introduction

Primary tumors of the heart are rare, occurring at a frequency of 0.001–0.03% based on autopsy studies [1, 2]. Cardiac papillary fibroelastomas (CPFEs) are historically known as the second most common primary cardiac tumors after cardiac myxomas. However, more recent studies suggest that CPFEs may be the most common cardiac tumor due to increasing diagnosis with echocardiography [3]. CPFEs consist of a small, papillary, pedunculated, and avascular tumor covered by endothelium surrounded by a hyaline stroma [4], though some authors believe that CPFEs are hamartomatous instead of neoplastic [5, 6]. Most of CPFEs are found on the valvular endocardium of the aortic and mitral valves, followed by the tricuspid and pulmonary valves and nonvalvular sites scattered in the atria and ventricles [7]. Multiple CPFEs can also form at the same location [3].

Although CPFEs are histologically benign and are usually asymptomatic, symptoms can occur when either the tumor itself or a thrombus embolizes. The most common clinical presentation is stroke or transient ischemic attack, followed by angina, myocardial infarction, sudden death, heart failure, syncope or presyncope, and systemic or pulmonary embolic events [7, 8]. Both transthoracic echocardiogram (TTE) and transesophageal echocardiogram (TEE) can be used to diagnose CPFE with high sensitivity and specificity [9]. On echocardiography, the tumor appears as a well-demarcated homogeneous round or oval structure and can appear “speckled” with stippling around the perimeter if the image quality is high [8, 9]. This report describes a case of a young male who presented with a cardioembolic stroke secondary to a CPFE diagnosed on TEE who was found to have *β*-thalassemia intermedia.

## 2. Case Presentation

The patient was a 29-year-old African American male without significant past medical history who presented to the emergency room with sudden weakness in his left lower extremity. While at work he suddenly felt lightheaded dizziness. Then, his left leg “gave out” while walking. Two days priorly, patient also had noted that his vision in his left eye suddenly became blurry, and his left hand clenched together uncontrollably and felt numb. Physical examination was normal except for mild loss of sensation in his left forearm. Troponin I was 0.196 and hemoglobin and MCV were 10.7 gm/dL and 70.8 fL, respectively. Urine drug screen was positive for cannabinoids. EKG revealed normal sinus rhythm with early repolarization changes. CT scan of the head without contrast showed loss of grey-white matter differentiation in the right insular cortex signifying a subacute ischemic stroke in the right middle cerebral artery distribution. He was started on aspirin, clopidogrel, and statin and was admitted for further workup.

The patient lacked significant cardiovascular risk factors that often cause ischemic stroke. He was young and normotensive and denied a family history of early cardiovascular disease. He smoked less than half a pack of cigarettes per day and used marijuana recreationally. He denied any IV drug use. He was generally active, with his most recent episode of prolonged immobilization being a six-hour car ride two weeks prior to presentation. He denied any past DVTs or a family history of hypercoagulability. The patient's neurological exam returned to normal within 48 hours of hospital admission.

**Figure 1:**
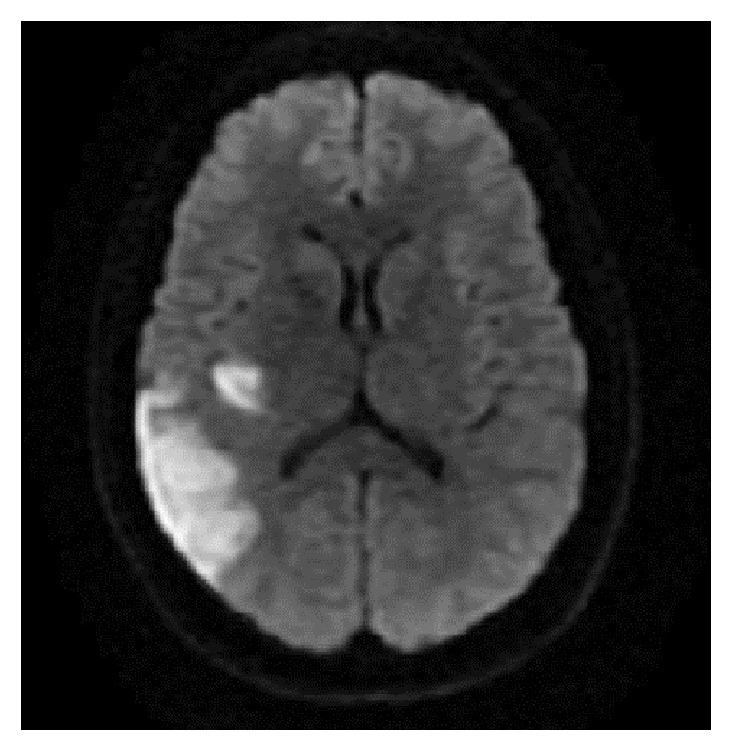
Axial images of diffusion-weighted MRI of the brain with contrast showing hyperintensity in the temporal and occipital lobes.

**Figure 2:**
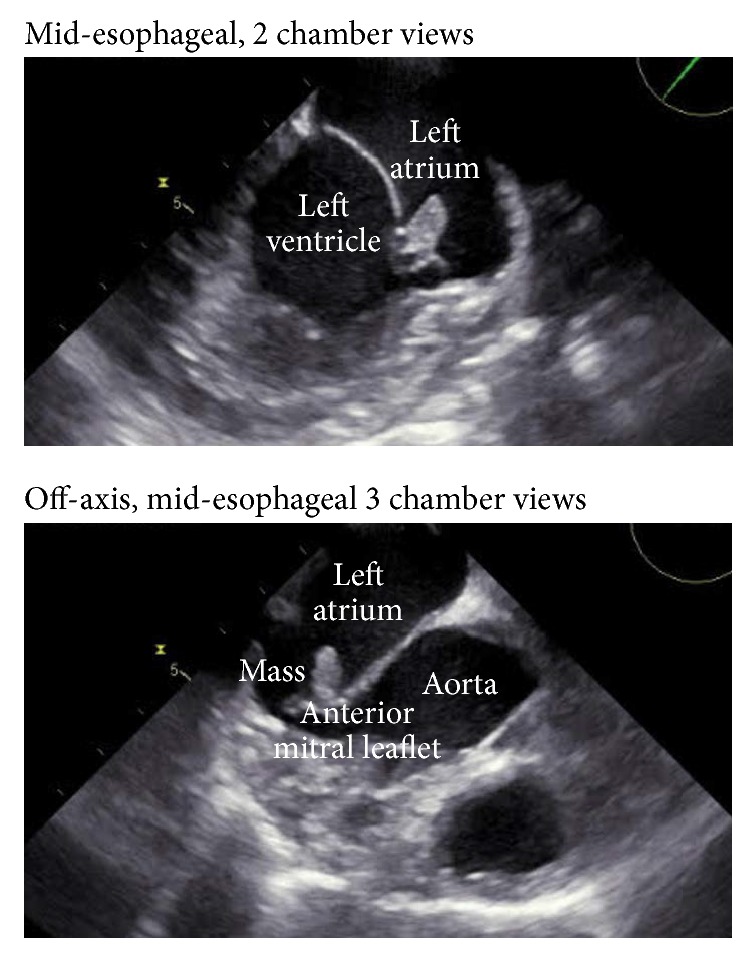
Transesophageal echocardiogram (TEE) image showing a pedunculated mass on the atrial aspect of the posterior leaflet of the mitral valve before surgical resection of the CPFE.

For stroke workup, patient underwent CT angiogram of the head and neck, which was normal. MRI of the brain with contrast showed abnormal signal in the right parietal lobe and other areas of small bright signals in the temporal lobe, occipital white matter, and right cerebellum consistent with multiple subacute infarcts (Figure 1). Transesophageal echocardiogram showed an 8 × 5 mm pedunculated mass on the atrial aspect of the posterior leaflet of the mitral valve (Figure 2), suggesting either a cardiac tumor or a vegetation of infectious or noninfectious etiology. Endocarditis was considered less likely due to lack of fever, along with four sets of negative blood cultures. In terms of the risk for endocarditis, the patient only met one minor Duke criterion, which was having a vascular phenomenon such as thromboembolic event.

**Figure 3:**
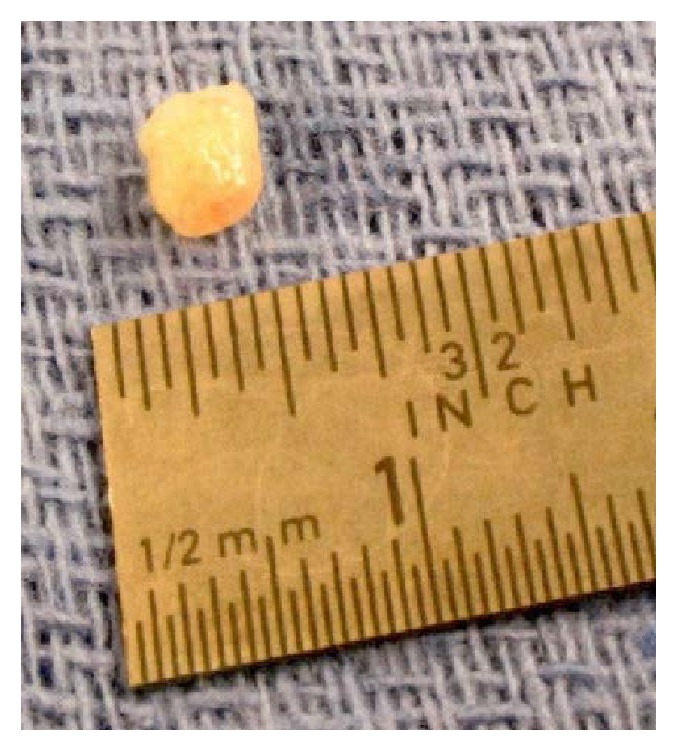
Surgical resection of the cardiac mass.

**Figure 4:**
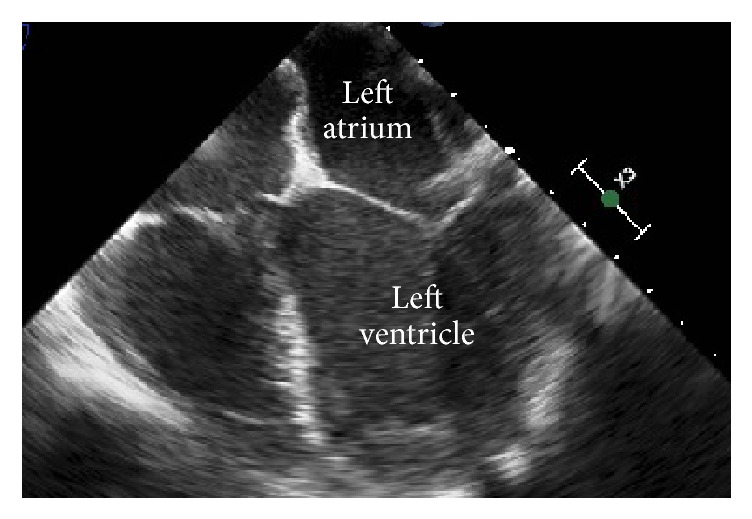
Transesophageal echocardiogram (TEE) image showing a preserved mitral valve after surgery resection of the CPFE.

**Figure 5:**
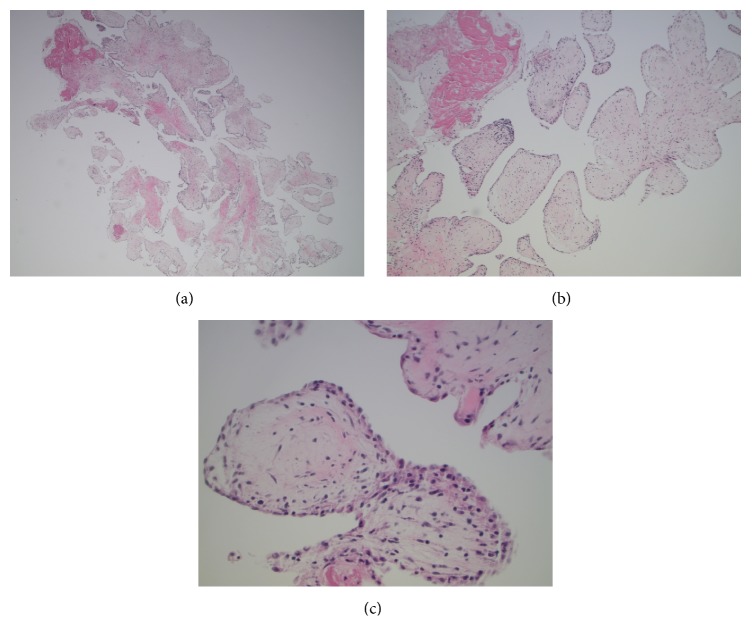
(a) H&E 40x reveals multiple papillary structures lined by endothelial cells; (b) H&E 100x reveals the papillary structures are avascular and contain fibromyxoid stroma; (c) H&E 400x reveals the papillary structures are lined by bland endothelial cells.

The patient was evaluated by cardiothoracic surgery and the lesion was resected three weeks later (Figure 3). Repeat TEE demonstrated the complete removal of the pedunculated mass (Figure 4). Pathology showed papillary structures lined by endothelium consisting of fibromyxoid stroma with dense areas of hyalinized stroma, confirming the diagnosis of a papillary fibroelastoma of the mitral valve (Figure 5). Fragments of this cardiac tumor were deemed the source of his cardioembolic ischemic stroke. After surgery, the patient was stable without further thromboembolic events or neurologic deficits. Hypercoagulable workup, including lupus anticoagulant, PT/PTT, anticardiolipin, homocysteine, and ANA, was negative. HIV serology was negative. Further workup of his microcytic anemia showed normal ferritin level of 197. Notably, on hemoglobin electrophoresis, Hgb A1 was decreased at 83.8% and Hgb F was increased at 13.2%, consistent with beta-thalassemia intermedia that was newly diagnosed.

## 3. Discussion

A stroke in a young and otherwise healthy individual without traditional risk factors presents an intriguing opportunity to investigate the cause of this clinical condition. Although CPFEs are rare and histologically benign, they should be managed with caution because they are a source of cardioembolic stroke [10]. Embolic strokes caused by arrhythmias and valvular diseases often require anticoagulation, but embolic strokes from CPFEs can be prevented through surgical resection of the tumor alone [8]. Surgical resection is the mainstay treatment and should be considered especially for CPFEs that are more than 10 mm in diameter, highly mobile, and associated with systemic embolization [11]. The outcomes following surgery are excellent: most valves retain their functionality and the tumors rarely recur [3]. Since CPFE has a curative management, understanding the pathogenesis of CPFE can guide disease diagnosis and prevent devastating neurological complications.

While CPFE is classified as a cardiac tumor, its pathogenesis differs significantly from that of other solid tumors and remains controversial. Instead of being driven by mutational changes and genomic instability, CPFEs are thought to arise from a combination of nongenetic causes: endothelial damage, viral-induced growth, microthrombi aggregation, and hamartomatous origin [7]. One of the more widely discussed theories suggests hemodynamic stress of blood flow causes damage to the endocardium, and continued hemodynamic stress over time leads to the accumulation of successive fibrin layers within the tumor [9]. Clinical studies support this hypothesis, showing that a large proportion of patients with CPFEs have associated cardiac and valvular conditions that increase hemodynamic stress [9]. The observation that CPFEs developed after iatrogenic stress to the endocardium through cardiac surgery and thoracic irradiation further supports this hypothesis [12].

More recently, a case report suggested a novel cause of damage to the endocardium in association with CPFEs in patient with *β*-thalassemia major [13]. *β*-Thalassemia, an inherited hemolytic disorder caused by the partial or complete inability to synthesize the *β*-chain in hemoglobin, is categorized into three groups based on the genetic alterations and clinical severity: major, intermedia, and minor. In the serum and plasma of patients with thalassemia, in vitro studies showed that there are elevated levels of endothelial adhesion proteins (intercellular adhesion molecule-1 [ICAM-1], E-selectin [ELAM-1], vascular cell adhesion molecule-1 [VCAM-1], von Willebrand factor [VWF], and thrombomodulin) [14, 15]. These endothelial adhesion proteins are elevated in the setting of endothelial activation and damage as well as chronic inflammation, suggesting these two processes underlie the pathophysiology of *β*-thalassemia [16]. These same processes, like hemodynamic stress, can also contribute to the formation of CPFEs.

In this case, this otherwise healthy patient did not have any cardiac or iatrogenic causes of increased hemodynamic stress. Based on literature review, this case is the second report that associates *β*-thalassemia with CPFE and is the first report specifically for *β*-thalassemia intermedia. While there is insufficient evidence to conclude whether his *β*-thalassemia contributed to the formation of the CPFE, this hypothesis-generating case calls attention to the need for elucidating the relationship between these two conditions in future studies.

## References

[1] Reynen K. (1996). Frequency of primary tumors of the heart. *The American Journal of Cardiology*.

[2] Centofanti P., Di Rosa E., Deorsola L. (1999). Primary cardiac tumors: early and late results of surgical treatment in 91 patients. *Annals of Thoracic Surgery*.

[3] Tamin S. S., Maleszewski J. J., Scott C. G. (2015). Prognostic and bioepidemiologic implications of papillary fibroelastomas. *Journal of the American College of Cardiology*.

[4] Butany J., Nair V., Naseemuddin A., Nair G. M., Catton C., Yau T. (2005). Cardiac tumours: diagnosis and management. *Lancet Oncology*.

[5] Dudley H. R., Goodale F., O'Neal R. M. (1956). Fibro-elastic hamartomas of heart valves. *The American Journal of Pathology*.

[6] Raeburn C. (1953). Papillary fibro-elastic hamartomas of the heart valves. *The Journal of Pathology and Bacteriology*.

[7] Gowda R. M., Khan I. A., Nair C. K., Mehta N. J., Vasavada B. C., Sacchi T. J. (2003). Cardiac papillary fibroelastoma: a comprehensive analysis of 725 cases. *American Heart Journal*.

[8] Sun J. P., Asher C. R., Yang X. S. (2001). Clinical and echocardiographic characteristics of papillary fibroelastomas: a retrospective and prospective study in 162 patients. *Circulation*.

[9] Klarich K. W., Enriquez-Sarano M., Gura G. M., Edwards W. D., Tajik A. J., Seward J. B. (1997). Papillary fibroelastoma: echocardiographic characteristics for diagnosis and pathologic correlation. *Journal of the American College of Cardiology*.

[10] Kasarskis E. J., William O., Earle G. (1988). Embolic stroke from cardiac papillary fibroelastomas. *Stroke*.

[11] Anastacio M. M., Moon M. R., Damiano R. J., Pasque M. K., Maniar H. S., Lawton J. S. (2012). Surgical experience with cardiac papillary fibroelastoma over a 15-year period. *Annals of Thoracic Surgery*.

[12] Kurup A. N., Tazelaar H. D., Edwards W. D. (2002). Iatrogenic cardiac papillary fibroelastoma: a study of 12 cases (1990 to 2000). *Human Pathology*.

[13] Kokotsakis J., Nenekidis I., Anagnostakou V. (2011). Papillary fibroelastoma of the aortic valve in a *β*-thalassemia patient. *General Thoracic and Cardiovascular Surgery*.

[14] Butthep P., Bunyaratvej A., Funahara Y. (1995). Alterations in vascular endothelial cell-related plasma proteins in thalassaemic patients and their correlation with clinical symptoms. *Thrombosis and Haemostasis*.

[15] Butthep P., Bunyaratvej A., Funahara Y. (1997). Possible evidence of endothelial cellactivation and disturbance in thalassemia: an in vitro study. *Southeast Asian Journal of Tropical Medicine and Public Health*.

[16] Kanavaki I., Makrythanasis P., Lazaropoulou C. (2009). Soluble endothelial adhesion molecules and inflammation markers in patients with *β*-thalassemia intermedia. *Blood Cells, Molecules, and Diseases*.

